# Image Charge Effects in the Wetting Behavior of Alkanes on Water with Accounting for Water Solubility

**DOI:** 10.3390/ma9030177

**Published:** 2016-03-08

**Authors:** Kirill A. Emelyanenko, Alexandre M. Emelyanenko, Ludmila B. Boinovich

**Affiliations:** A.N. Frumkin Institute of Physical Chemistry and Electrochemistry, Russian Academy of Sciences, Leninsky prospect 31 bldg. 4, 119071 Moscow, Russia; emelyanenko.kirill@gmail.com (K.A.E.); ame@phyche.ac.ru (A.M.E.)

**Keywords:** disjoining pressure, surface forces, contact angle, discrete charges, pentane, hexane, heptane, film stability, electrostatic interactions

## Abstract

Different types of surface forces, acting in the films of pentane, hexane, and heptane on water are discussed. It is shown that an important contribution to the surface forces originates from the solubility of water in alkanes. The equations for the distribution of electric potential inside the film are derived within the Debye-Hückel approximation, taking into account the polarization of the film boundaries by discrete charges at water-alkane interface and by the dipoles of water molecules dissolved in the film. On the basis of above equations we estimate the image charge contribution to the surface forces, excess free energy, isotherms of water adsorption in alkane film, and the total isotherms of disjoining pressure in alkane film. The results indicate the essential influence of water/alkane interface charging on the disjoining pressure in alkane films, and the wettability of water surface by different alkanes is discussed.

## 1. Introduction

The wetting of water by oils and the oil spreading on water are important fundamental and environmental issues, due to the significant increase in the area of the oil spill caused by spreading and its impact on the marine life and water. Considerable attention in recent studies was paid to experimental and theoretical studies on wetting of the surface of water and aqueous solutions by hydrocarbons and to the analysis of stability of alkane wetting films [1,2,3,4,5,6,7,8,9]. 

The experimentally-observed establishment of small but finite contact angle between the bulk meniscus of liquid C_6_–C_8_ alkanes and the water surface [6] can be theoretically explained on the basis of macroscopic theory of van der Waals forces. However, the existence of very thin, on the order of a few molecular diameters, wetting oil films in equilibrium with a droplet on a water surface contradicts to theoretical predictions, based on van der Waals forces. Earlier it was suggested in the literature [10] that, in some cases, the coexistence between the droplet and thin liquid film (the pseudo partial wetting) can be explained by invoking the short-range forces of a different nature, in addition to van der Waals forces. In our previous theoretical studies [2,3] we have shown that some of the peculiarities of wetting of water and brine solutions by alkanes from pentane to octane can be described by taking into account the image charge forces. The solubility of water in alkanes, although small, gives rise to the formation of a water-enriched adsorption layer at the interfaces of alkane films. The interaction of dipoles of water molecules in the adsorbed layer with their own images in confining phases and/or with discrete surface charges and their images contributes to the abovementioned image charge forces. The great importance of accounting the discreteness of surface charge was widely discussed in the literature when considering the electric potential distribution near charged interfaces, behavior of particles in electric fields, colloid aggregation, particle deposition, and adsorption phenomena [2,3,4,11,12,13,14,15,16,17,18,19,20,21]. Particularly, the polarization of alkane film boundaries by the electric field of dipoles of water dissolved in the film results in an appearance of image charge interaction between water molecules and film surfaces. Thus, the additional surface forces stabilizing the wetting film of alkane on water provide the coexistence between the wetting film and the sessile oil droplet on top of an aqueous surface. The analysis, presented in [2] considers the electrostatic energy of dissolved water molecules in the field of images of dipoles in confined phases. At the same time, charging of water/oil interface is well ascertained [11] and has to be taken into account in the analysis of excess alkane film energy associated with the image-charge forces. 

In this study we will present the theoretical analysis of surface forces, arising due to interaction of dipoles of water molecules, adsorbed in the hydrocarbon film, with both the polarized film boundaries and with charges at the water/alkane interface. The dependence of water monolayer adsorption on the film thickness for films of alkanes from pentane to heptane will be considered. Finally, we will discuss the role of weak water solubility in alkanes in the stability of alkane wetting films and in the wettability of aqueous phases by oils.

## 2. Excess Energy of Alkane Film Associated with Water Solubility in Alkane

As discussed above, the explanation of wetting behavior of low molecular weight alkane films on aqueous media requires invoking the surface forces components in addition to the van der Waals forces. It is reasonable to attribute the appearance of these additional forces to the solubility of water in alkanes. The analysis of the literature shows that, in contrast to the traditionally believed negligible amount of water in liquid hydrocarbons, the real bulk concentration reaches as much as a fraction of millimoles even in medium chain alkanes [22,23,24,25]. Additionally, the interaction of water molecules dissolved in thin film with the film boundaries by different types of interaction, including dispersion forces, hydrogen bonding, and image-charge forces, results in a further increase in water content in alkane wetting films. At the same time, the experimental data on the stability of thin wetting films and interlayers of solutions with a nonpolar solvent and polar solute indicate the high responsivity of equilibrium thickness to the presence of polar molecules [26,27,28,29].

Several mechanisms of image-charge forces, associated with polarization of phases confining the film in the electric field of dipoles of solute polar molecules have to be considered. Some of them were analyzed in our previous works [2,18]. Namely, we have derived the equations for calculation of excess free energy of the film, including the energy of dipoles of solute molecules in the dipole image-charge field, taking into account the variable orientation of dipoles relative to the surface normal and their disordering along the interface. The interaction of dipole monolayers adsorbed on the opposite film surfaces via image-charge interaction and the respective contribution to the film energy was calculated as well. At the same time, the charging of water/alkane interface leads to essential electrostatic fields inside the wetting film, additional polarization of film boundaries, and affects both the adsorption of water molecules and their energy in the oil film which have to be taken into account. In the next section we will discuss how the electrostatic potential and electric field intensity, induced by discrete charges at the interface, are distributed across the film.

### 2.1. Electrostatic Potential in Alkane Film Induced by a Discretely Charged Interface, with Charges, Located in the Electrolyte Phase Close to the Interface

**Figure 1:**
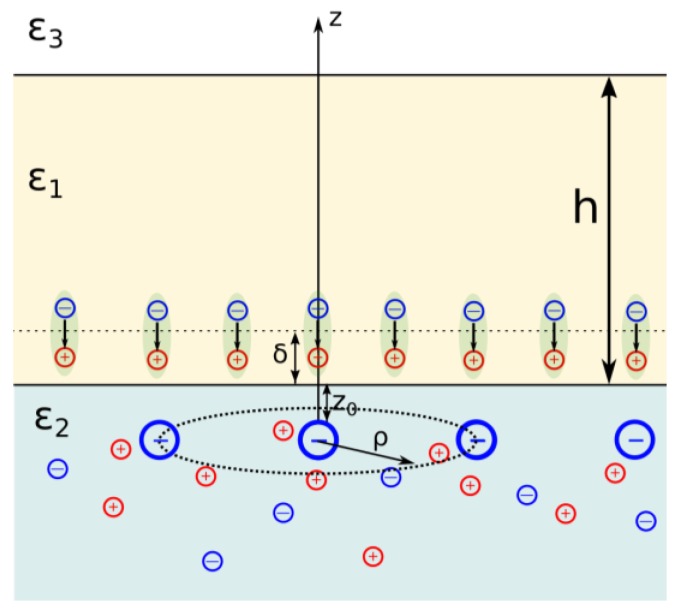
The cylindrical coordinate system coupled with a point charge.

The model system, containing the wetting film of nonpolar liquid with dielectric permittivity *ε*_1_ and thickness *h*, confined by two semi-infinite media with dielectric permittivities *ε*_2_ and *ε*_3_, is depicted in Figure 1.

Medium 2 represents an aqueous electrolyte solution, whereas Media 1 and 3 are dielectric ones. The point charge *q*, appearing due to charging of electrolyte/dielectric interface, is located within electrolyte medium at distance *z*_0_ from the position of the interface. We will consider the induced electrostatic fields in the cylindrical coordinate system with the origin coinciding with the charge position and *Z* axis normal to the interface (Figure 1). The arrangement of surface charges is not ordered due to ions’ thermal motion. The charge induces, on one hand, the polarization of the interfaces, and, on the other hand, the formation of a diffuse ionic atmosphere of electrolyte ions in the electrolyte solution. The dipoles with a dipole moment *p*, appearing due to the solubility of water in alkanes, are located within the alkane wetting film at a distance δ from the position of the interface and induce the polarization of the interfaces as well. The dipole position and orientation in the film is mainly defined by hydrogen bonding between the dissolved water molecule and aqueous substrate (the medium 2). The relative arrangement of dipoles in the film and charges in the electrolyte cannot be defined unambiguously because, as it will be shown below, the energy of their interaction is less than the characteristic energy of thermal motion, *k_B_T* (where *k_B_* is the Boltzmann constant and *T* is the absolute temperature).

Due to the superposition principle the electric potential distribution in the contacting media is defined as a sum of the potentials induced by different electric sources, such as the surface ions and their images or the dipoles of dissolved water molecules and their images. The distribution of potentials in contacting media, induced by each type of electric source, was obtained on the basis of simultaneous solution of the Poisson equations in three contacting media, as presented in [2,3,18].

For water or diluted 1-1 electrolyte aqueous solutions, when inverse Debye length *κ* << 1/*z*_0_, the potential at arbitrary point z, ρ in the film, induced by a single point charge *q* and its image charges can be written within the Debye-Hückel approximation as [3]:

(1) φ(z,ρ)=2qε2∑k=0∞(−β13)k[1(ρ2+(z+2kh)2)12+β13(ρ2+(z−2z0−2(k+1)h)2)12]

where $\beta_{13}=\left(\varepsilon_{1}-\varepsilon_{3}\right)/\left(\varepsilon_{1}+\varepsilon_{3}\right)$ . Note, that the Equation (1) is accurate for lateral distances ρ<<1/κ .

To calculate the potential created by electric charges, discretely distributed in a plane, and keeping in mind that the thermal motion results in the smoothing of the discontinuous distribution of charges, it is efficient to use the method of a cut-out disk [30,31,32]. In this model, the charge density around the chosen point charge is approximated by a step function:

(2) $$\sigma_{q}(\rho )=\{\begin{array}{cc} 0, & 0<\rho <\rho_{0,q}\\ \sigma =q/\pi \rho_{0,q}^{2}, & \rho_{0,q}<\rho <\infty \end{array}$$

where $\pi \rho_{0,q}^{2}$ is the average area per surface charge at water-oil interface. Consequently, the potential, $\varphi_{\Sigma ,q}$ , of the electric field induced by all the real surface charges, and by all the image charges, at the location defined by radius-vector *r*, where $\left|r\right|=\sqrt{z^{2}+\rho^{2}}$ , is expressed as a sum of contributions from a discrete charge and a plane of smeared charge with a hole. In other words, the potential, $\varphi_{\Sigma ,q}$ , is contributed by the discrete charge and uniformly-charged plane less the disc with a center at charge location:

(3) $$\varphi_{\Sigma ,q}(z,\rho )=\varphi_{0}(z,\rho )+\varphi_{disk}(z,\rho )+\varphi_{plane}(z,\rho )$$

where $\varphi_{0}$ is the potential of the field induced by a given charge *q* and all its images, $\varphi_{disk}$ is the potential, induced by an uniformly-charged disk of the radius $\rho_{0,q}$ with a total charge equal to *–q* and all disk images and, finally, $\varphi_{plane}$ represents the potential induced by an uniformly charged plane with a surface charge density *σ* and all images of this plane.

The potential due to first two terms in the right hand part of Equation (3) has the form, dependent on the ratio $r_{k}/\rho_{0,q}$ (where *r_k_* is the distance between the point with coordinates *(z,ρ)* and *k*th image). In the case when $r_{k}>\rho_{0,q}$ we get:

(4) $$\varphi_{0}(z,\rho )+\varphi_{disk}(z,\rho )=\frac{4q}{\varepsilon_{2}}\sum_{k=0}^{\infty }{\left(-\beta_{13}\right)}^{k}\sum_{m=1}^{\infty }\frac{P_{2m+2}(0)}{2m+1}\rho_{0,q}^{2m}\times \{\frac{P_{2m}\left(\frac{z+2kh}{\sqrt{\rho^{2}+{\left(z+2kh\right)}^{2}}}\right)}{{\left(\sqrt{\rho^{2}+{\left(z+2kh\right)}^{2}}\right)}^{2m+1}}+\frac{\beta_{13}P_{2m}\left(-\frac{z-2z_{0}-2(k+1)h}{\sqrt{\rho^{2}+{\left[z-2z_{0}-2(k+1)h\right]}^{2}}}\right)}{{\left(\sqrt{\rho^{2}+{\left[z-2z_{0}-2(k+1)h\right]}^{2}}\right)}^{2m+1}}\}$$

while in the opposite case of $r_{k}<\rho_{0,q}$ :

(5) $$\begin{array}{c} \varphi_{0}(z,\rho )+\varphi_{disk}(z,\rho )=\frac{2q}{\varepsilon_{2}}\sum_{k=0}^{\infty }{\left(-\beta_{13}\right)}^{k}\cdot \{\frac{1}{\sqrt{\rho^{2}+{\left(z+2kh\right)}^{2}}}+\frac{2\sqrt{\rho^{2}+{\left(z+2kh\right)}^{2}}}{\rho_{0,q}^{2}}\\ -\sum_{m=0}^{\infty }\frac{2P_{2m}(0)}{1-2m}\frac{{\left(\sqrt{\rho^{2}+{\left(z+2kh\right)}^{2}}\right)}^{2m}}{\rho_{0,q}^{2m+1}}P_{2m}\left(\frac{z+2kh}{\sqrt{\rho^{2}+{\left(z+2kh\right)}^{2}}}\right)\\ +\frac{\beta_{13}}{\sqrt{\rho^{2}+{\left[z-2z_{0}-2(k+1)h\right]}^{2}}}+\frac{2\beta_{13}\sqrt{\rho^{2}+{\left[z-2z_{0}-2(k+1)h\right]}^{2}}}{\rho_{0,q}^{2}}\\ -\sum_{m=0}^{\infty }\frac{2P_{2m}(0)}{1-2m}\frac{\beta_{13}{\left(\sqrt{\rho^{2}+{\left[z-2z_{0}-2(k+1)h\right]}^{2}}\right)}^{2m}}{\rho_{0,q}^{2m+1}}P_{2m}\left(\frac{-\left[z-2z_{0}-2(k+1)h\right]}{\sqrt{\rho^{2}+{\left[z-2z_{0}-2(k+1)h\right]}^{2}}}\right)\} \end{array}$$

In Equations (4) and (5), *P_n_*(*x*) is the *n*th-degree Legendre polynomial. It is worth noting that contributions of different images for the same charge should be calculated on the basis of appropriate equation, Equation (4) or Equation (5), depending on the distance between the given point and this image. 

For $r_{k}=\rho_{0,q}$ it is easy to show that Equations (4) and (5) give the same result. Using the Equations (4) and (5), the components of electric field intensity induced by discrete charges at water-oil interface and their images were derived.

For $r_{k}>\rho_{0,q}$ the *z*-component of electric field intensity is expressed as:

(6) $$E_{z}(z,\rho )=\frac{4q}{\varepsilon_{2}}\sum_{k=0}^{\infty }{\left(-\beta_{13}\right)}^{k}\sum_{m=1}^{\infty }P_{2m+2}(0)\rho_{0,q}^{2m}\times \left(\frac{P_{2m+1}\left(\frac{z+2kh}{\sqrt{\rho^{2}+{\left(z+2kh\right)}^{2}}}\right)}{{\left(\sqrt{\rho^{2}+{\left(z+2kh\right)}^{2}}\right)}^{2m+2}}+\beta_{13}\frac{P_{2m+1}\left(\frac{\left[z-2z_{0}-2(k+1)h\right]}{\sqrt{\rho^{2}+{\left(z+2kh\right)}^{2}}}\right)}{{\left(\sqrt{\rho^{2}+{\left[z-2z_{0}-2(k+1)h\right]}^{2}}\right)}^{2m+2}}\right)$$

where it was taken into account that, due to electro neutrality of electrolyte solution, the double electric layer constituted by an uniformly-charged interface and a diffuse ion layer performs as a capacitor with zero electric field intensity outside:

(7) $$E_{z,plane}=0$$

For $r_{k}<\rho_{0,q}$ the *z*-component of electric field intensity is expressed as:

(8) $$\begin{array}{c} E_{z}(z,\rho )=\frac{2q}{\varepsilon_{2}}\sum_{k=0}^{\infty }{\left(-\beta_{13}\right)}^{k}\times \{\frac{\left(z+2kh\right)}{{\left(\sqrt{\rho^{2}+{\left(z+2kh\right)}^{2}}\right)}^{3}}-\frac{2}{\rho_{0,q}^{2}}\frac{\left(z+2kh\right)}{\sqrt{\rho^{2}+{\left(z+2kh\right)}^{2}}}\\ +\sum_{m=0}^{\infty }\frac{2P_{2m}(0)}{1-2m}\frac{2m{\left(\sqrt{\rho^{2}+{\left(z+2kh\right)}^{2}}\right)}^{2m-1}}{\rho_{0,q}^{2m+1}}P_{2m-1}\left(\frac{z+2kh}{\sqrt{\rho^{2}+{\left(z+2kh\right)}^{2}}}\right)\\ +\frac{\beta_{13}\left[z-2z_{0}-2(k+1)h\right]}{{\left(\sqrt{\rho^{2}+{\left[z-2z_{0}-2(k+1)h\right]}^{2}}\right)}^{3}}-\frac{2\beta_{13}}{\rho_{0,q}^{2}}\frac{\left[z-2z_{0}-2(k+1)h\right]}{\sqrt{\rho^{2}+{\left[z-2z_{0}-2(k+1)h\right]}^{2}}}\\ +\beta_{13}\sum_{m=0}^{\infty }\frac{2P_{2m}(0)}{1-2m}\frac{2m{\left(\sqrt{\rho^{2}+{\left[z-2z_{0}-2(k+1)h\right]}^{2}}\right)}^{2m-1}}{\rho_{0,q}^{2m+1}}P_{2m-1}\frac{\left[z-2z_{0}-2(k+1)h\right]}{\sqrt{\rho^{2}+{\left[z-2z_{0}-2(k+1)h\right]}^{2}}}\} \end{array}$$

We have also derived the equations for *ρ*-component (parallel to the interface) of the electric field intensity. It was found that the modulus of *E_ρ_* is a small quantity, and the dipole oriented parallel to the interface at any location within the film with *ρ ≠ 0* will have the electrostatic energy in the field of discretely charged plane much less than *k_B_T*. That leads to free rotation of parallel dipoles in alkane film around *z*-axis and hence to zero average contribution of parallel dipoles of solute to the excess free energy of the wetting film. As for the parallel dipoles located at *ρ* = *0*, their energy is equal to zero, since $E_{\rho }(z,\rho =0)\equiv 0$ . Therefore, using the Equations (6) and (8) we now can calculate the electrostatic contribution to the excess free energy of the film, arising from the interaction of solute polar molecules with the discretely charged aqueous medium-oil interface, as:

(9) $$U_{q}=-\vec{p}{\vec{E}}_{z}=-pE_{z}\cos\theta$$

where *θ* is the angle constituted by the dipole moment of solute molecule with the direction of the *z*-component of electric field intensity induced by discrete charges at the interface and infinite series of their images in the phases confining the film.

### 2.2. Image-Charge Contribution to the Excess Adsorption of Solute Water Molecules and to the Excess Free Energy of Alkane Wetting Films on Water

Total excess free energy of the alkane wetting film may be considered as a sum of contributions of the van der Waals forces, electrostatic image charge forces [33], and the excess energy of hydrogen bonding interaction of solute water molecules with aqueous substrate. As we will show below, the most significant energy contribution per each dipole of solute water, which essentially exceeds the *k_B_T*, is related to the hydrogen bonding. This is why the state of water molecules in the alkane film can be considered as an adsorbed state at water-alkane interface. It is important to note that two different type of sources lead to the appearance of image-charge forces. Namely, as it was mentioned above, both the electric field of discrete charges at the interface and the electric field of dipoles of solute water cause the polarization of film boundaries. Due to the superposition principle the consideration of the total image charge energy as a sum of independent energy contributions arising from different electric sources (dipoles or charges) is quite accurate.

In this approach we ignore the interaction of charges with their images (considered earlier in [3]) as well as the interactions between dipole images and charge images. However, the numerical calculations show that typically these contributions are extremely low and we can omit them from our consideration.

So, for the excess free energy of wetting film, U , we have:

(10) $$U=U_{123}+\bar{U}\cdot \Gamma^{\left(12\right)}=U_{123}+\Gamma^{\left(12\right)}\cdot (U_{q}+U_{dip}+U_{OH}+U_{vdW})$$

where $U_{123}$ is the energy of the van der Waals interactions of film boundaries through the alkane interlayer; Г^(12)^ is the number of water molecules per unit of film area, adsorbed on the water-alkane interface; $U_{q},U_{dip},U_{OH},U_{vdW}$ are the electrostatic energies per one dipole for the dipole in the field of discrete charges and their images, the electrostatic energy for the dipole in the field of other dipoles and their images, the energy of hydrogen bonding between the water molecule and aqueous phase, and the energy of the van der Waals interaction of adsorbed water molecules with phases confining the film, respectively.

The results of a molecular dynamics study [34] of the structure of liquid in the vicinity of a water–hydrocarbon interface showed that near the interface water molecules presented in the alkane film will be preferably oriented in such a manner that one O–H bond was normal to the interface. The second O–H bond can experience free rotation around the first one. Such configuration provides a maximum energy of hydrogen bonding between the water molecules in the alkane interlayer and the aqueous substrate and corresponds to one hydrogen bond per adsorbed water molecule ( $U_{OH}\approx -8.6k_{B}T$ ). In this case the total dipole moment of a water molecule makes an angle of about 52° with the normal to the interface. For the sake of simplicity we will consider two components of the solute water dipole moment, one of them being parallel and the other normal to the interface.

As it was shown in [4], the electrostatic energy of a dipole in the field of other dipoles and their images is represented by the relation:

(11) $$U_{dip}=U_{im}^{n}+U_{im}^{p}+U_{lat}$$

where $U_{im}^{n},U_{im}^{p}$ are the potential energies of the dipoles oriented normal and parallel to the interface in the field of dipole own images, respectively; $U_{lat}$ is the potential energy of a dipole in the electric field induced by other dipoles adsorbed in the monolayer and their images. The relations derived earlier in [2,4,18] were used to calculate the values of $U_{im}^{n},\;U_{im}^{p}$ and $U_{lat}$ .

For calculating the energy of the van der Waals interaction, $U_{vdW}$ , between the adsorbed water molecule and the phases two and three, confining the alkane film, the following equation is valid [35]:

(12) $$U_{vdW}(\delta )=\frac{-3k_{B}Tv_{m}}{8\pi \delta^{3}}\sum_{N=0}^{\infty }\frac{\varepsilon_{w}(i\xi_{N})-\varepsilon_{1}\left(i\xi_{N}\right)}{\varepsilon_{w}(i\xi_{N})+2\varepsilon_{1}(i\xi_{N})}\;\frac{\varepsilon_{2}(i\xi_{N})-\varepsilon_{1}(i\xi_{N})}{\varepsilon_{2}(i\xi_{N})+\varepsilon_{1}(i\xi_{N})}-\frac{3k_{B}Tv_{m}}{8\pi {\left(h-\delta \right)}^{3}}\sum_{N=0}^{\infty }\frac{\varepsilon_{w}(i\xi_{N})-\varepsilon_{1}\left(i\xi_{N}\right)}{\varepsilon_{w}(i\xi_{N})+2\varepsilon_{1}(i\xi_{N})}\;\frac{\varepsilon_{3}(i\xi_{N})-\varepsilon_{1}(i\xi_{N})}{\varepsilon_{3}(i\xi_{N})+\varepsilon_{1}(i\xi_{N})}$$

Here ν*_m_* is the water molecular volume, and $\varepsilon_{w}(i\xi )$ is the imaginary frequency dependence of dielectric permittivity of water, $\varepsilon_{j}(i\xi )$ is the imaginary frequency dependence of the dielectric permittivity for water (*j* = 2), alkane (*j* = 1), or vapor (*j* = 3), and δ was accepted to be equal to half the water monolayer height [36], *δ* = 0.13 nm.

Based on Equations (6), (8)–(12), and using the dielectric data of water and alkanes, measured in [37], we have calculated the contributions of different nature into the total adsorption potential for water molecule in 1 nm thick alkane film. In the Table 1 the data for pentane and hexane films are presented. The electrostatic energy of dipole in the field of discretely charged interface and polarized film boundaries is sensitive to the mutual arrangement of a given dipole and the closest discrete charge (see Equations (6), (8) and (9)). According to [11], the surface charge density characteristic of water-oil interface is −5 to −7 μC/cm^2^, which roughly corresponds to one hydroxide ion on every 3 nm^2^ of the surface (*ρ*_0,*q*_ = 1 nm). Thus, for the density of dipoles in the adsorbed monolayer higher than the density of charges, the maximum distance *ρ* between the charge and the dipole will not exceed 1 nm. To illustrate the effect of mutual arrangement of the dipole and the charge, we present in Table 1 the data for two different lateral positions (defined by *ρ*), corresponding to the dipole location exactly above the point charge (*ρ* = 0) and above the edge of “cut-out” disk (*ρ* = *ρ*_0,*q*_).

**Figure 2:**
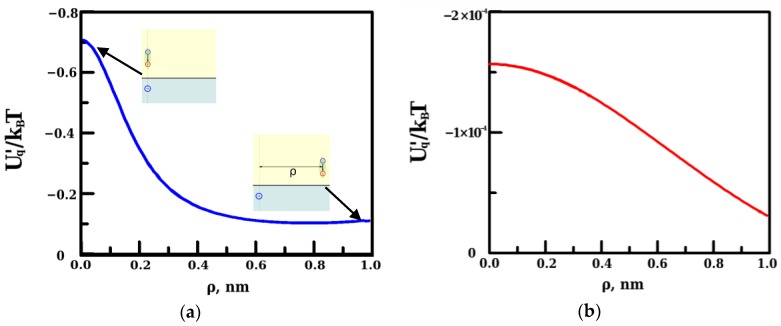
(**a**) The energy of interaction between the dipole of solute water molecule and a discretely charged water/alkane interface with accounting for all polarization effects, as a function of dipole location with respect to a given surface charge; and (**b**) the thickness dependent part of the energy presented in (**a**).

The analysis of data in Table 1 shows, that all types of adsorption potentials, with the exception of the energy of lateral interactions with all dipoles adsorbed in the monolayer and their images, promote an increase in the adsorption of water molecules in the alkane film. The main contribution to the adsorption potential is related to the energy of hydrogen bonding. However, this potential does not cause the dipole spatial ordering in the monolayer. In contrast, the energy of lateral interaction tends to push the dipoles out from the adsorbed monolayer and supports a spatially uniform distribution of dipoles. As for the interaction of a dipole with a discrete charge, its energy is less than *k_B_T*, even in the case of the closest mutual arrangement “dipole just above the charge” and quickly decays with an increase in relative distance *ρ* (Figure 2a). It is worth noting that the magnitude of this energy is mainly defined by the interaction between the real surface charge and the dipole. However, this contribution is independent of film thickness (see Equations (6) and (8)). As for the thickness dependent part of the energy, which is related to the interaction between the dipole and the images of real charge, its magnitude is three orders of magnitude less (Figure 2b).

To calculate the adsorption in the water monolayer inside the film we followed the approach developed in [2,18]. The Langmuir-type isotherm with adsorption potential $\bar{U}$ , presented in Equation (10), was used:

(13) $$\begin{array}{c} \Gamma^{\left(12\right)}=\Gamma_{0}^{\left(12\right)}\frac{\exp(-\bar{U}/k_{B}T)x}{1+\exp(-\bar{U}/k_{B}T)x} \end{array}$$

where *x* is a molar portion of water solution in alkane, which in our calculations was considered equal to the solubility limit of water in a given alkane at 20 °C (for water in pentane *x* = 3.3 × 10^−4^; for water in hexane *x* = 5.0 × 10^−4^; for water in heptane *x* = 5.0 × 10^−4^); $\Gamma_{0}^{\left(12\right)}$ is the number of adsorption sites per unit of interfacial area, which was accepted, following [34], to be equal to 7.1 nm^−2^. 

**Figure 3:**
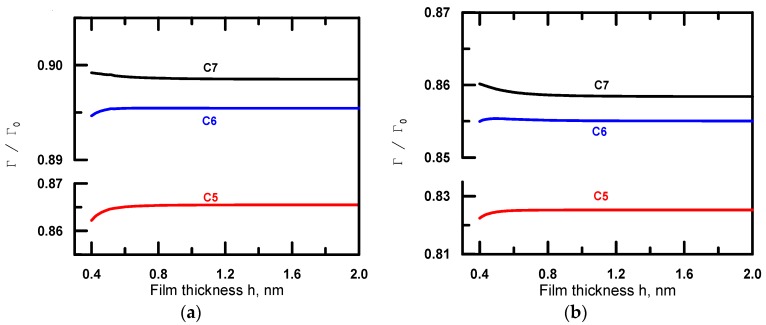
Adsorption isotherms of water in alkane films from pentane to heptane calculated for two different mutual locations of a dipole and a charge: (**a**) for *ρ* = 0; and (**b**) for *ρ* = *ρ_0,q_* = 1 nm.

The adsorption isotherms versus alkane film thickness for alkanes C_5_–C_7_ are presented in Figure 3 for two different mutual locations of a dipole and a charge. This data indicate that for three considered alkanes the surface coverage is close to complete monolayer. Nevertheless, it is affected by the type of alkane, on one hand, due to the variation in dipole-dipole interaction energy and on the other hand, due to changes in the energy of the van der Waals interaction between the water molecule and confining phases. The water content in alkane film turns out to be the thickness dependent. It decreases upon thickness increase for pentane and decreases for heptane films.

## 3. Image-Charge Contribution to the Disjoining Pressure in Alkane Wetting Films on Water and its Influence on the Wettability

According to the definition [38] and basing on Equation (10), the total magnitude of the disjoining pressure can be calculated as a sum of the van der Waals component of disjoining pressure and the electrostatic component associated with image charge interactions of different types:

(14) $$\Pi =-\frac{dU}{dh}=\Pi_{vdW}+\Pi_{image}$$

where

(15) $$\Pi_{image}=-\frac{d\bar{U}}{dh}=-\frac{d\Gamma^{\left(12\right)}}{dh}(U_{OH}+U_{vdW}+U_{dip}+U_{q})-\Gamma^{\left(12\right)}\frac{d(U_{vdW}+U_{dip}+U_{q})}{dh}$$

**Figure 4:**
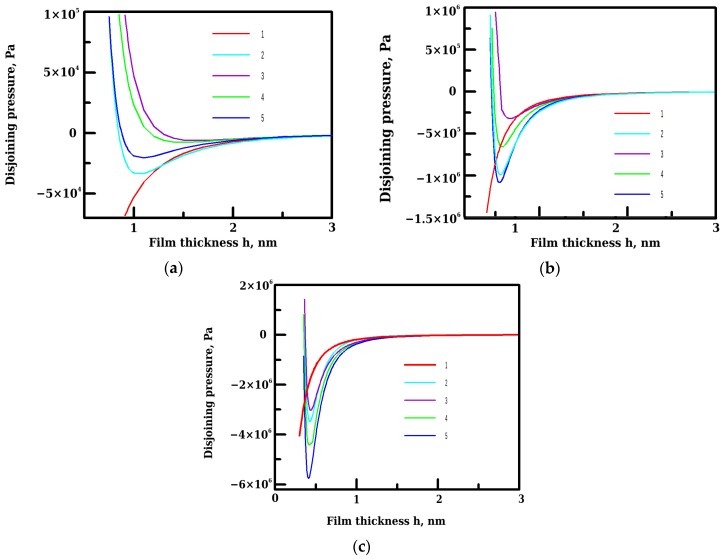
Calculated isotherms of the disjoining pressure for wetting films of: (**a**) pentane; (**b**) hexane; and (**c**) heptane. On each panel, five types of curves are given, corresponding to the van der Waals component of disjoining pressure (1), to total isotherm when the image forces were estimated for *σ* = 0 (2), and to total isotherms with image forces estimated for *σ* = –5 μC·cm^−2^ and water dipoles located at *ρ* = 0 (3), at *ρ* = 0.5 nm (4), and at *ρ* = 1 nm (5).

We have calculated the isotherms of disjoining pressure for films of pentane, hexane and heptane on water surface for zero surface charge and for charge density σ = –5 μC·cm^−2^, typical for water-oil interfaces [11]. The first case corresponds to accounting for the image forces induced by the interaction of dipoles of solute water molecules with other dipoles in the film and their images. For the latter case, the location of dipoles relative to the charge is important. The analysis of Equations (6) and (8) show that the maximum contribution to image-charge forces, associated with the interaction of dipoles with surface charges, corresponds to the location of dipoles just above the charge, whereas the location of dipole at *ρ* = *ρ_0,q_* leads to the minimum in the absolute value of П_image_. The energy of interaction of dipole with discretely charged interface and the correspondent images is less than *k_B_T* and essentially less than $U_{lat}$ . Therefore, the Boltzman-type distribution degenerates to a spatially uniform one with random locations of the dipoles with respect to the charge. Additionally, the surface density of charges does not coincide with the density of dipoles in the monolayer. The latter, as may be concluded from the surface coverage (see Figure 3), is essentially higher. Thus, we can give the estimation of П_image_ as being in between the isotherms of П_image_ calculated under conditions that (1) all dipoles are located at *ρ* = *0* and (2) all dipoles are located at *ρ* = *ρ_0,q_*. The isotherms of the disjoining pressure calculated for wetting films of pentane, hexane, and heptane on water are presented in Figure 4a–c. On each graph, five types of curves are given, corresponding to the van der Waals component of disjoining pressure (curve 1), to total isotherm when the image forces were estimated for *σ* = 0 (curve 2), and to total isotherms with image forces estimated for *σ* = –5 μC·cm^−2^ and water dipoles located at *ρ* = 0 (curve 3), at *ρ* = 0.5 nm (curve 4), and at *ρ* = 1 nm (curve 5). 

The analysis of the presented isotherms shows that including the image charge forces into consideration allows predicting theoretically the existence of stable alkane films with the thickness *h_0_* at coexistence with the sessile droplets. Such films were observed experimentally in [6]. In the case of macroscopic sessile droplet, the thickness of such films is defined on the stable branch of the disjoining pressure isotherm at the point П(*h*) = 0 where the total isotherm intersects the thickness axis. Thus, for pentane film this thickness, depending on above mentioned conditions of calculations, varied from 0.88 to 1.3 nm (see Table 2) indicating the formation of polymolecular wetting films of pentane in equilibrium with the droplet of pentane on water. In contrast, the equilibrium thickness of heptane films varied from 0.35 to 0.37 nm indicating the formation of an adsorption monolayer rather than wetting the film of heptane in equilibrium with an alkane droplet, with a carbon backbone lying parallel to interface.

On the basis of the calculated isotherms of total disjoining pressure it is possible now to apply the Derjaguin and Frumkin theory of wetting [38] for estimating the equilibrium contact angles *θ* formed by alkane droplets on water surface:

(16) $$\sigma_{LV}\cdot \cos\theta =\sigma_{LV}+\int_{h_{0}}^{\infty }\Pi (h)dh$$

where $\sigma_{LV}$ is the surface tension of the corresponding alkane. The contact angles, calculated according to Equation (16) at the conditions mentioned above are presented in Table 2.

The results of calculations indicate that for pentane and hexane films the action of image charge forces induced by interaction of dipoles with discretely charged interface leads to improvement of wetting. In heptane film, the tendency is opposite; charging of the interface causes an increase in the contact angle in comparison to that, calculated at the conditions of zero surface charge, when the only mechanism of image-charge forces is related to the interaction of dipoles of solute molecules with other dipoles in the film and their images. Such different behavior results from the variation of dielectric parameters of alkanes in the series C_5_–C_7_ leading to the different thickness dependence of water adsorption in alkane film (Figure 3).

It was interesting to estimate the influence of surface charge density on the thicknesses of alkane films coexisting with the droplet on the water surface and on the droplet contact angle. We have performed the calculations for the average lateral distance between the surface charge and a dipole of solute water molecule. It is easy to show by simple algebra, taking into account the random locations of the dipoles with respect to the charge (as discussed above), that the average lateral distance is equal to (2/3)*ρ*_0*,q*_*.*

**Figure 5:**
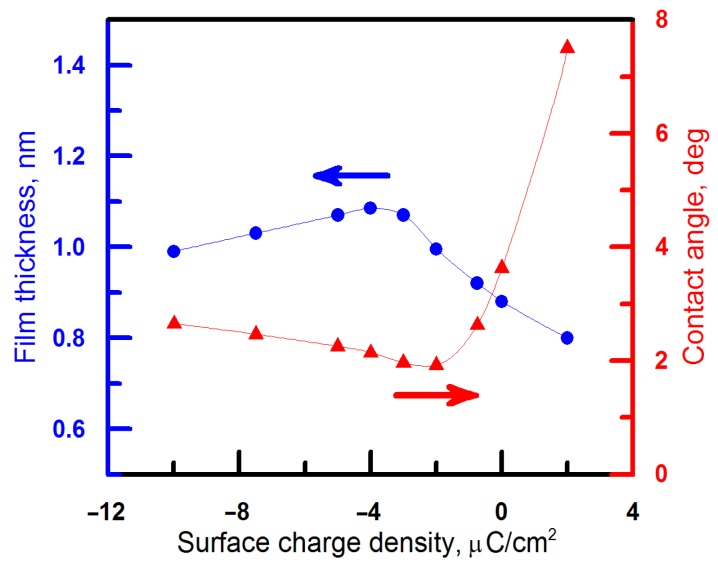
Calculated dependences of pentane wetting film thicknesses and of the contact angle formed by the pentane droplet on the surface of the aqueous phase *versus* the surface charge density.

The results of calculations presented in Figure 5 indicate non-monotonic dependence with low sensitivity of pentane film thickness to the surface charge density and *h*_max_ = 1.1 nm at *σ* = 4 μC·cm^−2^. The contact angle formed by the pentane droplet on the surface of aqueous phase varies non-monotonically with the surface charge density as well, with more significant sensitivity to the sign of surface charge. 

## 4. Concluding Remarks

The explanation of experimentally-detected peculiarities of wetting behavior of low molecular weight alkanes on water requires invoking the surface force components additional to the van der Waals forces. It was reasonable to associate the mechanism of these additional forces with the presence of water dissolved in alkane films. The interaction of solute water molecules with each other, with the discretely charged water–alkane interface, and with polarized film boundaries causes the appearance of the film thickness dependent excess free energy of the film. In this study we have described the above polarization and interactions on the basis of concept of image-charge forces. The presented theoretical analysis, performed within the Debye-Hückel approximation allowed us to derive equations for the distribution of electric potential and electric field intensity inside the film. It was shown that the excess free energy arising due to interaction of dipoles of water molecules dissolved in the hydrocarbon film with the charges at water–alkane interface and with their images leads to the stabilization of wetting/adsorption alkane films. The comparison of the total isotherms of disjoining pressure in the films of alkanes from C_5_ to C_7_ indicates the significant role of the variation of dielectric parameters of alkanes with chain length. Such variation causes the different thickness dependence of water adsorption in alkane films. Additionally, the analysis of the presented isotherms of the disjoining pressure shows that including the image charge forces into consideration allows predicting, theoretically, the experimentally-observed phenomena. Namely, the existence of stable alkane films at coexistence with the sessile droplets and the increase in contact angle in the series C_5_–C_7_ are well reproduced.

## Figures and Tables

**Table 1:** Contributions of different types of interactions into the total adsorption potential for water molecules in 1 nm thick alkane film.

System	*ρ*, nm	*U_OH_*/*k_B_T*	*U_disp_*/*k_B_T*	*U_im_^n^*/*k_B_T*	*U_im_^P^*/*k_B_T*	*U_lat_*/*k_B_T*	*U_q_*/*k_B_T*
water/pentane/air	0.0	−8.6	−0.88	−1.84	−1.54	3.70	−0.70
	1.0	−8.6	−0.88	−1.84	−1.54	3.45	−0.10
water/hexane/air	0.0	−8.6	−0.92	−1.80	−1.51	3.78	−0.70
	1.0	−8.6	−0.92	−1.80	−1.51	3.56	−0.11

**Table 2:** Equilibrium film thicknesses and the contact angles, calculated using Equation (16) and isotherms of the disjoining pressure presented in Figure 4, for films of different alkanes on a water surface.

Alkane	for *σ* = 0		for *σ* = –5 μC·cm^−2^					
			*ρ* = 0		*ρ* = 0.5 nm		*ρ* = 1 nm	
	*h*_0_, nm	*θ*, °	*h*_0_, nm	*θ*, °	*h*_0_, nm	*θ*, °	*h*_0_, nm	*θ*, °
Pentane	0.88	3.6	1.30	2.0	1.2	2.1	0.88	2.9
Hexane	0.47	12.1	0.56	8.1	0.48	10.2	0.46	12.3
Heptane	0.37	16.9	0.37	17.2	0.36	19.4	0.35	22.3

*ρ*, location of water dipoles with respect to charge.
